# *Bombyx mori*‐Hosted *Cordyceps militaris* Polysaccharides: Structural Characterization and Antibacterial Activity of Native and Sulfated Derivatives

**DOI:** 10.1002/cbdv.71798

**Published:** 2026-09-30

**Authors:** Jingchun Yang, Jiani Cheng, Gaoyang Li, Wenpu Shi, Yaqi Liu, Tingyu Luo, Lixue Zheng

**Affiliations:** ^1^ School of Biology and Food Engineering Suzhou University of Technology Changshu Jiangsu China; ^2^ School of Traditional Chinese Materia Medica Shenyang Pharmaceutical University Shenyang Liaoning China; ^3^ Suzhou Xinfudi Agricultural Products Market Development Co., Ltd. Changshu Jiangsu China

**Keywords:** antibacterial activity, *Bombyx mori*‐hosted *Cordyceps militaris*, polysaccharides, sulfated modification, structural characterization

## Abstract

Polysaccharides from *Bombyx mori*‐hosted *Cordyceps militaris* are abundant but exhibit limited bioactivity, necessitating exploration of sulfated derivatives. In this study, crude polysaccharides (Bm‐CMP) were extracted and further sulfated to obtain four derivatives (Bm‐sCMP‐1∼4). Physicochemical and structural analyses indicated that sulfation significantly modified the characteristics of Bm‐CMP, notably reducing the molecular weight (Mw) and particle size while enhancing the negative surface charge and altering the monosaccharide profile. All sulfated derivatives exerted enhanced inhibitory effects against *Escherichia coli* and *Bacillus subtilis*, which were closely dependent on the degree of substitution (DS), sulfate content, and negative charge density. Notably, Bm‐sCMP‐2, with a moderate DS, displayed the optimal antibacterial efficacy. Collectively, this work demonstrates that sulfation is a feasible strategy to valorize polysaccharides from *B. mori*‐hosted *C. militaris*, transforming them into promising candidates for novel antibacterial agents, although their in vivo efficacy and safety require further validation.

## Graphical abstract

This study demonstrates that sulfation enhances the antibacterial activity of *Bombyx mori*‐hosted *Cordyceps militaris* polysaccharides. Activity correlated positively with sulfate content and negative charge density, with the highest substituted derivative exhibiting optimal efficacy. These findings validate sulfation as a feasible strategy to valorize these polysaccharides into novel antibacterial agents.

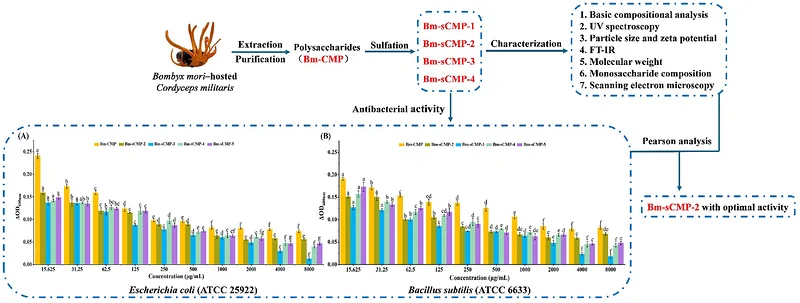

## 1 Introduction

Foodborne pathogens pose a critical global challenge to food safety and public health. According to the World Health Organization (WHO), foodborne illnesses affect approximately 600 million people and cause 420,000 deaths annually worldwide, with the highest burden observed in the Southeast Asia and Eastern Mediterranean regions [1]. To mitigate microbial growth and extend shelf life, the food industry has relied heavily on synthetic preservatives such as sodium benzoate, potassium sorbate, and nitrites [2, 3]. Despite these measures, pathogen contamination in ready‐to‐eat foods, dairy, meat, and aquatic products persists as a critical issue, posing ongoing risks to consumer health and incurring substantial socioeconomic costs [4, 5]. Consequently, current research prioritizes the discovery of novel antimicrobial agents that combine high efficacy with safety, biodegradability, and natural origins.

In recent years, natural polysaccharides have attracted considerable interest as renewable and biocompatible antimicrobial agents [6, 7, 8]. However, their intrinsically weak activity necessitates structural modification [9]. Sulfation, a strategy that introduces negatively charged sulfate groups to enhance electrostatic interactions with bacterial membranes, has become a widely validated approach for improving antibacterial efficacy [10, 11]. This has been demonstrated in studies on various polysaccharides, including those from fungi. Specifically, sulfated derivatives of *β*‐glucans from *Ganoderma lucidum*, as well as polysaccharides from *Antrodia cinnamomea* and cultured *Cordyceps militaris* fruiting bodies, all exhibit significantly improved antimicrobial, anticoagulant, or antitumor activities compared to their native forms [12, 13, 14, 15]. However, most of these reports focus on polysaccharides derived from conventional mycelium or fruiting‐body cultures on synthetic or grain‐based substrates [16]. In sharp contrast, the sulfation modification and bioactivity of polysaccharides from insect‐hosted *Cordyceps* species, a unique cultivation system with distinct nutritional composition and potentially different polysaccharide structures, have received limited attention and remain insufficiently explored [17, 18].

*Bombyx mori* is a well‐established medicinal insect characterized by mature breeding protocols and cost‐effective production [19]. Compared with conventional mycelium‐based *Cordyceps*, *B. mori*‐hosted *C. militaris* is richer in proteins, chitin, and specialized lipids, and the nutrient composition of the growth substrate can further influence the bioactivity of the resulting *Cordyceps* [20]. Furthermore, it contains significantly higher levels of polysaccharides and cordycepin than *C. militaris* on synthetic substrates [21]. However, the antibacterial potential of native polysaccharides from this host system is not well understood, and the effects of sulfation modification on their activity have not been systematically investigated.

Therefore, the objective of this study was to prepare sulfated polysaccharides from *B. mori*‐hosted *C. militaris* using the sulfamic acid method. Their structural characteristics and in vitro antibacterial activity were preliminarily investigated, aiming to lay a foundation for subsequent in‐depth structural elucidation and comprehensive bioactivity evaluation.

## 2 Results and Discussion

### 2.1 Physicochemical Properties

Hot‐water extraction yielded 28.95 ± 3.32% crude polysaccharides from *B. mori*‐hosted *C. militaris*. Following purification, the resulting polysaccharides (Bm‐CMP) exhibited a high carbohydrate content of 75.21 ± 4.84% and a minimal sulfate content of 1.48 ± 0.26% (Table 1). Following sulfation modification, the sulfate content in Bm‐CMP derivatives (Bm‐sCMP‐1∼4) increased significantly, peaking at 13.45 ± 0.36% for Bm‐sCMP‐2 (*p* < 0.01). Correspondingly, the degree of substitution (DS) increased from 0.078 ± 0.014 to 1.193 ± 0.056 in Bm‐sCMP‐2 (*p* < 0.01). However, further increasing the mass ratio of SA to polysaccharide led to a decrease in DS (*p* < 0.05). This may be attributed to the excessively acidic conditions of the reaction system, which induce degradation of the polysaccharide chains. In addition, an excess of reagents may impede effective contact between the reactants, leading to an inhomogeneous reaction [22, 23]. Additionally, compared to native Bm‐CMP, the carbohydrate contents and protein contents in Bm‐sCMP‐1∼4 were significantly reduced (*p* < 0.05 or *p* < 0.01). This finding is in accordance with previous research, suggesting that it may be a consequence of the increasing proportion of sulfate groups [24].

**TABLE 1:** The physicochemical properties of Bm‐CMP and Bm‐sCMP‐1∼4.

Fraction	Bm‐CMP:SA (mg/mg)	Carbohydrates (%)	Protein (%)	Uronic acids (%)	Sulfates (%)	DS	Zeta potential (mV)	Particle size (nm)	Mw (kDa)	Monosaccharide composition
Bm‐CMP	1:0	75.21 ± 4.84^a^	3.21 ± 0.17^a^	5.08 ± 0.32^a^	1.48 ± 0.26^d^	0.078 ± 0.014^d^	−13.3 ± 1.35^c^	593.7 ± 11.3^a^	2494.83 (1: 3.8%) 3.23 (2: 96.2%) Average: 48.96	Arab (1.85%), GlcN (0.66%), Gal (3.53%), Glc (85.15%), Xyl (2.37%), Man (4.98%), Rib (1.46%).
Bm‐sCMP‐1	1:2	68.34 ± 4.14^a^	0.32 ± 0.07^c^	3.97 ± 0.12^b^	5.74 ± 0.52^c^	0.356 ± 0.040^c^	−14.6 ± 0.87^c^	275.2 ± 4.56^b^	7.76 (1: 100%)	Arab (6.77%), GlcN (0.17%), Gal (13.73%), Glc (50.07%), Xyl (7.41%), Man (21.32%), GalA (0.52%).
Bm‐sCMP‐2	1:3	50.52 ± 2.62^b^	0.17 ± 0.03^d^	1.77 ± 0.10^e^	13.45 ± 0.36^a^	1.193 ± 0.056^a^	−24.9 ± 1.13^a^	48.52 ± 9.34^d^	5.43 (1: 100%)	Arab (1.52%), GlcN (0.5%), Gal (15.02%), Glc (55.6%), Xyl (3.01%), Man (23.65%), GalA (0.7%).
Bm‐sCMP‐3	1:4	46.30 ± 1.46^b^	0.43 ± 0.09^b^	2.08 ± 0.13^d^	12.71 ± 0.91^ab^	1.086 ± 0.129^ab^	−18.6 ± 0.95^b^	56.67 ± 25.36^cd^	3.93 (1:100%)	Arab (6.31%), GlcN (0.27%), Gal (12.37%), Glc (56.69%), Xyl (6.62%), Man (23.65%).
Bm‐sCMP‐4	1:5	45.51 ± 1.06^b^	0.45 ± 0.12^b^	2.60 ± 0.06^c^	11.72 ± 0.74^b^	0.949 ± 0.098^b^	−14.7 ± 0.54^c^	91.14 ± 31.24^c^	6.04 (1: 100%)	Arab (5.7%), Gal (11.13%), Glc (58.75%), Xyl (6.75%), Man (17.67%).

^a^ *Abbreviations*: Bm‐CMP, *B. mori*‐hosted *C. militaris* polysaccharides; Bm‐sCMP‐1∼4, different sulfated Bm‐CMP derivatives; DS, degree of substitution; Mw, molecular weight; Arab, arabinose; GlcN, Glucosamine; Gal, galactose; Glc, glucose; Xyl, xylose; Man, mannose; GalA, galacturonic acid; Rib, Ribose.

^b^Different lowercase letters superscripts in the same column indicate statistically significant differences (*p* < 0.05 or *p* < 0.01).

Uronic acids are widely recognized as key determinants of the net negative charge in polysaccharides [25]. In this study, despite a significant reduction in uronic acid content across Bm‐sCMP‐1∼4 following sulfation (*p* < 0.01), all sulfated derivatives exhibited markedly more negative zeta (*ζ*) potentials compared to native Bm‐CMP (*p* < 0.01). This shift correlated positively with the DS, indicating that the enhanced negative surface charge of the sulfated derivatives was predominantly attributed to the introduced sulfate groups rather than uronic acids [26].

As shown in Table 1 and Figure 1, the molecular weight (Mw) of Bm‐CMP and Bm‐sCMP‐1∼4 were determined to be 48.96, 7.76, 5.43, 3.93, and 6.04 kDa, respectively. Chromatographic profiles revealed that while native Bm‐CMP comprised two distinct molecular fractions, the sulfated derivatives exhibited a single symmetric peak, suggesting a more homogeneous Mw distribution. Notably, a significant reduction in Mw was observed after sulfation, consistent with previous reports demonstrating that sulfation processes often induced partial depolymerization of polysaccharides due to acidic conditions or thermal degradation [27]. Furthermore, this phenomenon was corroborated by particle size analysis, which revealed a significant decrease in the particle size as the SA ratio increased (*p* < 0.05 or *p* < 0.01), consistent with the general dependence of hydrodynamic volume on Mw [28].

**FIGURE 1:**
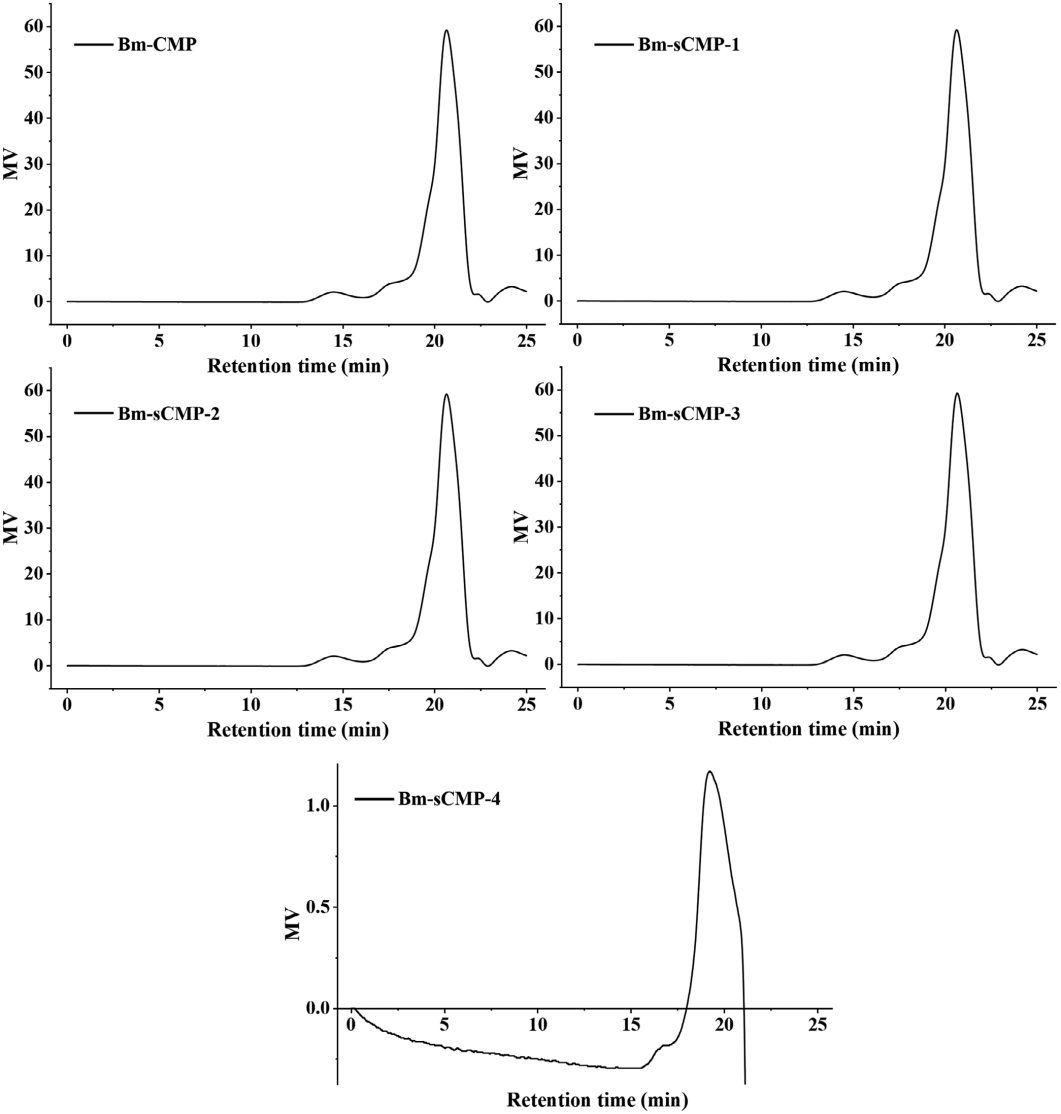
The Mw distributions of Bm‐CMP, Bm‐sCMP‐1∼4. Bm‐CMP, *B. mori*‐hosted *C. militaris* polysaccharides; Bm‐sCMP‐1∼4, different sulfated Bm‐CMP derivatives.

The monosaccharide composition of Bm‐CMP and Bm‐sCMP‐1∼4 was shown in Figure 2. Monosaccharide types and proportions were identified by comparison with authentic standards based on retention times. As summarized in Table 1, native Bm‐CMP consisted primarily of glucose (85.15%), along with mannose (4.98%), galactose (3.53%), xylose (2.37%), arabinose (1.85%), ribose (1.46%), and glucosamine (0.66%). Although Bm‐sCMP‐1 and Bm‐sCMP‐2 exhibited identical monosaccharide profiles, the compositions of Bm‐CMP, Bm‐sCMP‐3, and Bm‐sCMP‐4 differed from each other. Nevertheless, all samples were predominantly composed of glucose, followed by mannose and galactose as minor components. Sulfation modification led to a decrease in the proportion of glucose, which was consistent with the physicochemical characterization and correlated with the DS. These results indicated that sulfation not only altered the relative proportions of constituent monosaccharides but also changed their compositional profile in certain derivatives. This suggested that the sulfation process may induce structural modifications affecting both the backbone and side chains of Bm‐CMP, and it also confirms the hydrolysis of polysaccharides under acidic conditions [29].

**FIGURE 2:**
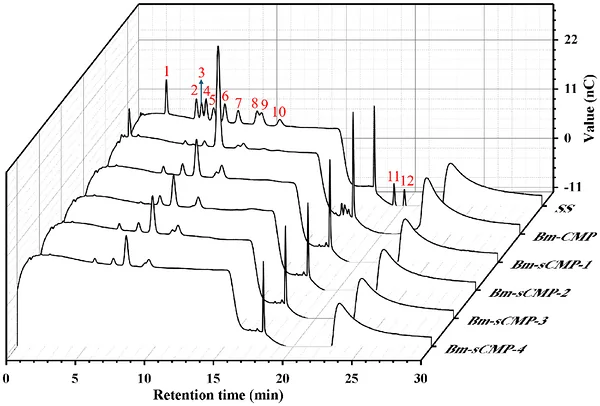
The ion chromatograms of monosaccharides in Bm‐CMP and Bm‐sCMP‐1∼4. Abbreviation: SS‐standard sugars. Peaks 1∼12 represent fucose, galactosamine, rhamnose, arabinose, glucosamine, galactose, glucose, xylose, mannose, ribose, galacturonic acid, and glucuronic acid, sequentially. Bm‐CMP, *B. mori*‐hosted *C. militaris* polysaccharides; Bm‐sCMP‐1∼4, different sulfated Bm‐CMP derivatives.

### 2.2 Spectral Analysis

The ultraviolet (UV) spectra were presented in Figure 3. All samples exhibited a strong peak at 200 nm, which is the characteristic UV absorption peak of polysaccharides. Meanwhile, weak peaks appeared at approximately 280 nm, the characteristic UV absorption wavelength of proteins, in both Bm‐CMP and the sulfated derivatives [30]. This observation was consistent with the results of the general component analysis (Table 1). Further investigation is required to determine whether the trace protein components exist as unpurified free protein or are covalently bound to the polysaccharides.

**FIGURE 3:**
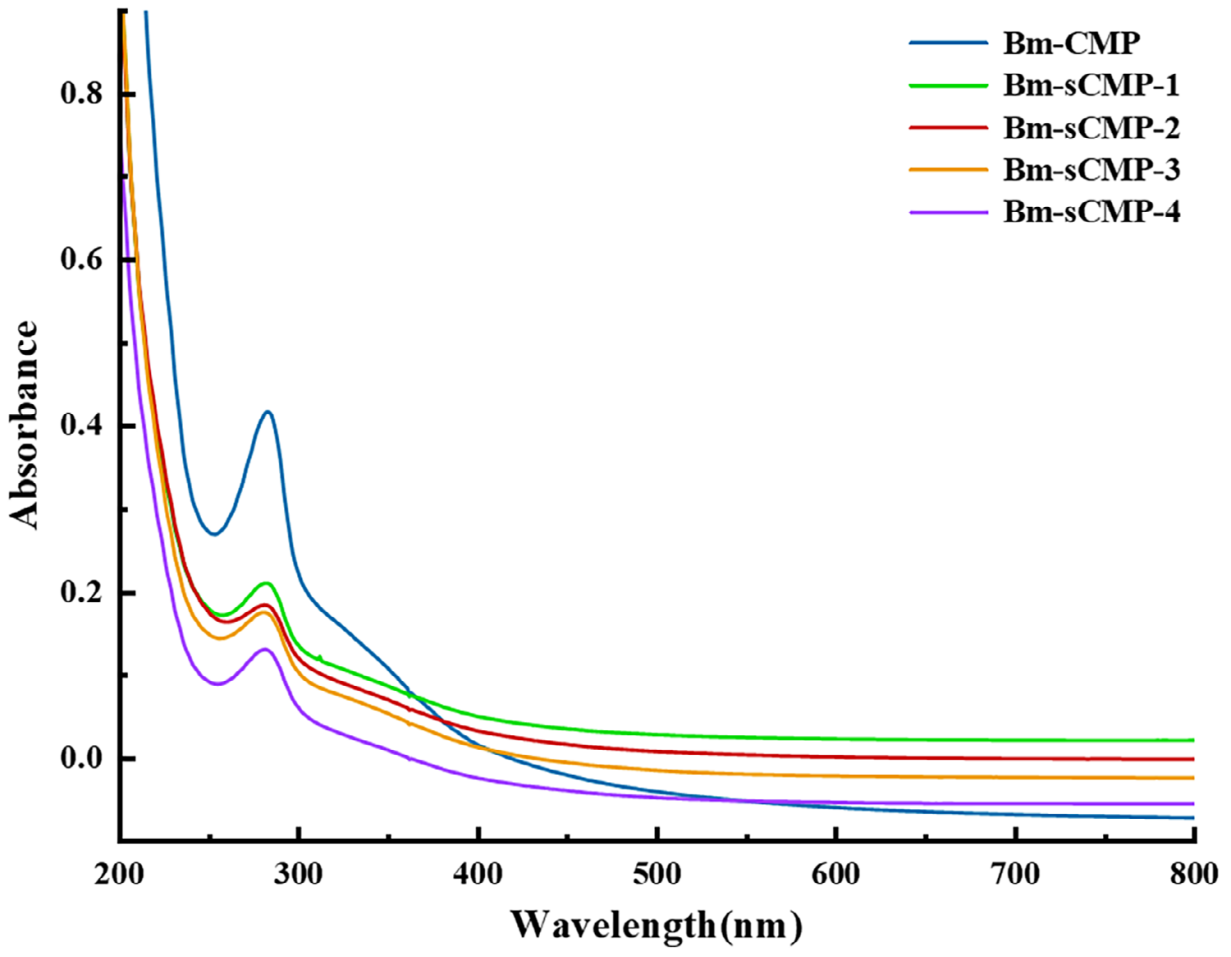
The UV spectra of Bm‐CMP and sulfated derivatives. Bm‐CMP, *B. mori*‐hosted *C. militaris* polysaccharides; Bm‐sCMP‐1∼4, different sulfated Bm‐CMP derivatives.

The FT‐IR spectra of Bm‐CMP and Bm‐sCMP‐1∼4 were presented in Figure 4. All samples displayed characteristic polysaccharide absorption bands near 3450, 2930, 1650, and 1399 cm^−1^. Specifically, the broad peak at approximately 3450 cm^−^^1^ was attributed to the O─H stretching vibration [31]; the band at 2930 cm^−^^1^ corresponded to C─H stretching vibrations [32]; the absorption at 1650 cm^−^^1^ was assigned to C═O stretching [33]; and the signal at 1399 cm^−^^1^ might arise from symmetric C─O stretching or methylene C─H bending vibrations [34]. Collectively, these features confirmed the polysaccharide nature of all the samples [35]. Distinct absorption peaks at 1150, 1080, and 1037 cm^−^^1^ were indicative of pyranose ring structures, suggesting that both Bm‐CMP and its sulfated derivatives were primarily composed of pyranose‐type monosaccharides, which were consistent with the monosaccharide composition analysis [36]. Notably, after sulfation, new characteristic bands appeared in Bm‐sCMP‐1∼4: a strong peak at 1260 cm^−^^1^, assigned to the asymmetric stretching vibration of S═O in sulfate groups; a band at 810 cm^−^^1^, corresponding to C─O─S stretching; and a signal at 582 cm^−^^1^, attributed to the bending vibration of S═O [37, 38]. As these signals were absent in the native Bm‐CMP spectrum, the FT‐IR data provided clear spectroscopic evidence for the successful introduction of sulfate groups into the polysaccharide backbone.

**FIGURE 4:**
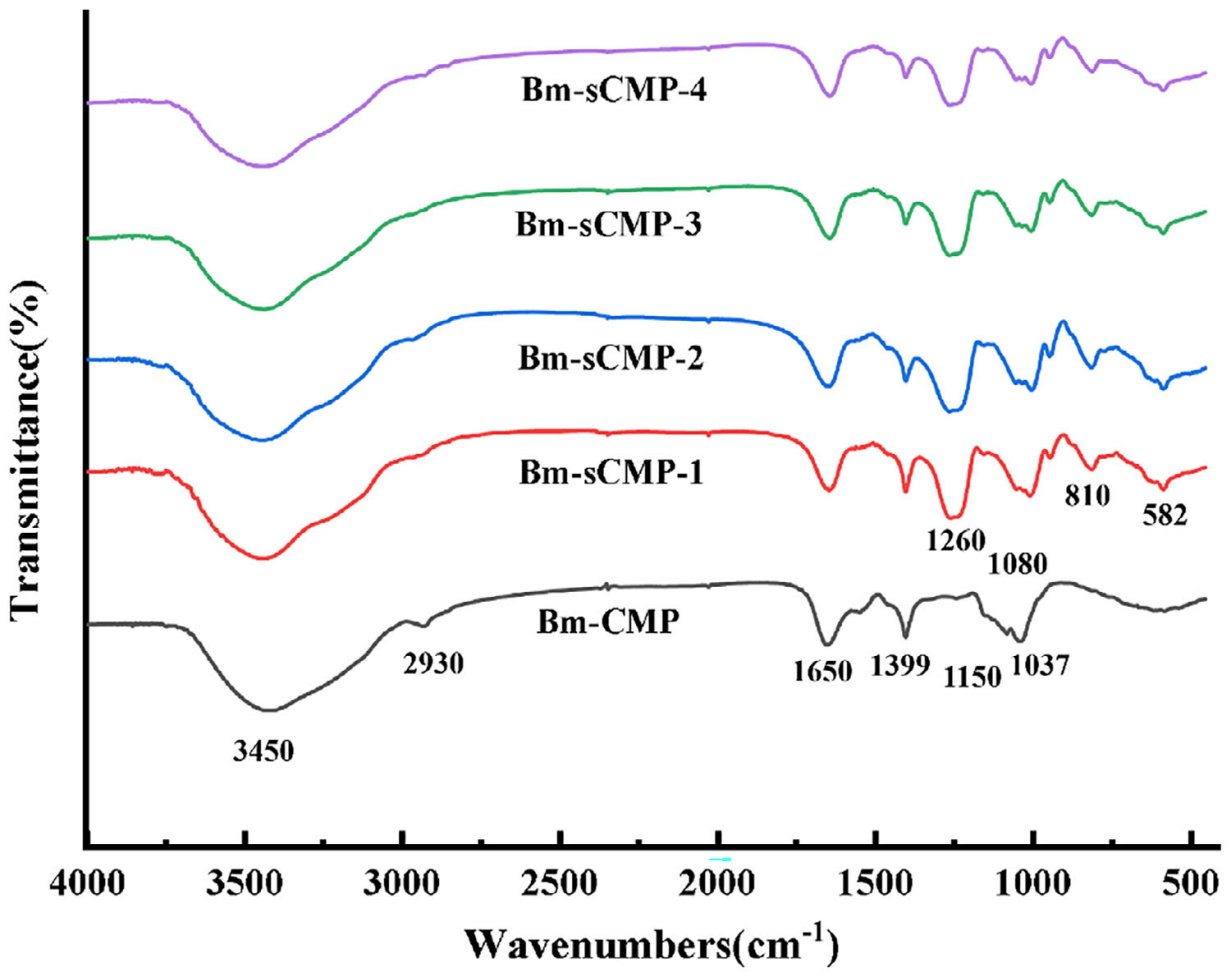
The FT‐IR spectra of Bm‐CMP and sulfated derivatives in the range of 4000–400 cm^−1^. Bm‐CMP, *B. mori*‐hosted *C. militaris* polysaccharides; Bm‐sCMP‐1∼4, different sulfated Bm‐CMP derivatives.

### 2.3 Morphological Analysis

Scanning electron microscopy (SEM) was employed to characterize the surface morphology of the polysaccharides. As illustrated in Figure 5, Bm‐CMP exhibited predominantly smooth, spherical particles with a relatively uniform distribution. In contrast, after sulfation, Bm‐sCMP‐1∼4 displayed distinct morphological alterations, characterized by increased aggregation and the emergence of diverse structural motifs, such as laminar sheets, rod‐like structures, and irregularly arranged small spheres. Notably, the extent of aggregation correlated with the DS, being most pronounced in Bm‐sCMP‐2, thereby corroborating the successful sulfation. It is generally accepted that the compactness of the polysaccharide surface observed in SEM images is positively correlated with Mw [39]. However, an intriguing phenomenon was observed in this study: despite a reduction in Mw (Table 1), all sulfated derivatives exhibited more extensive aggregation compared to native Bm‐CMP, accompanied by a clear transition from spherical to sheet‐like and rod‐shaped morphologies. This seemingly paradoxical phenomenon might be attributed to the introduction of sulfate groups, which effectively facilitated the cross‐linking and aggregation of short molecular chains [40]. Therefore, to comprehensively elucidate the surface properties of sulfated polysaccharides, it was essential to conduct further investigations into the interactions among other physicochemical properties and structural parameters of the polysaccharides.

**FIGURE 5:**
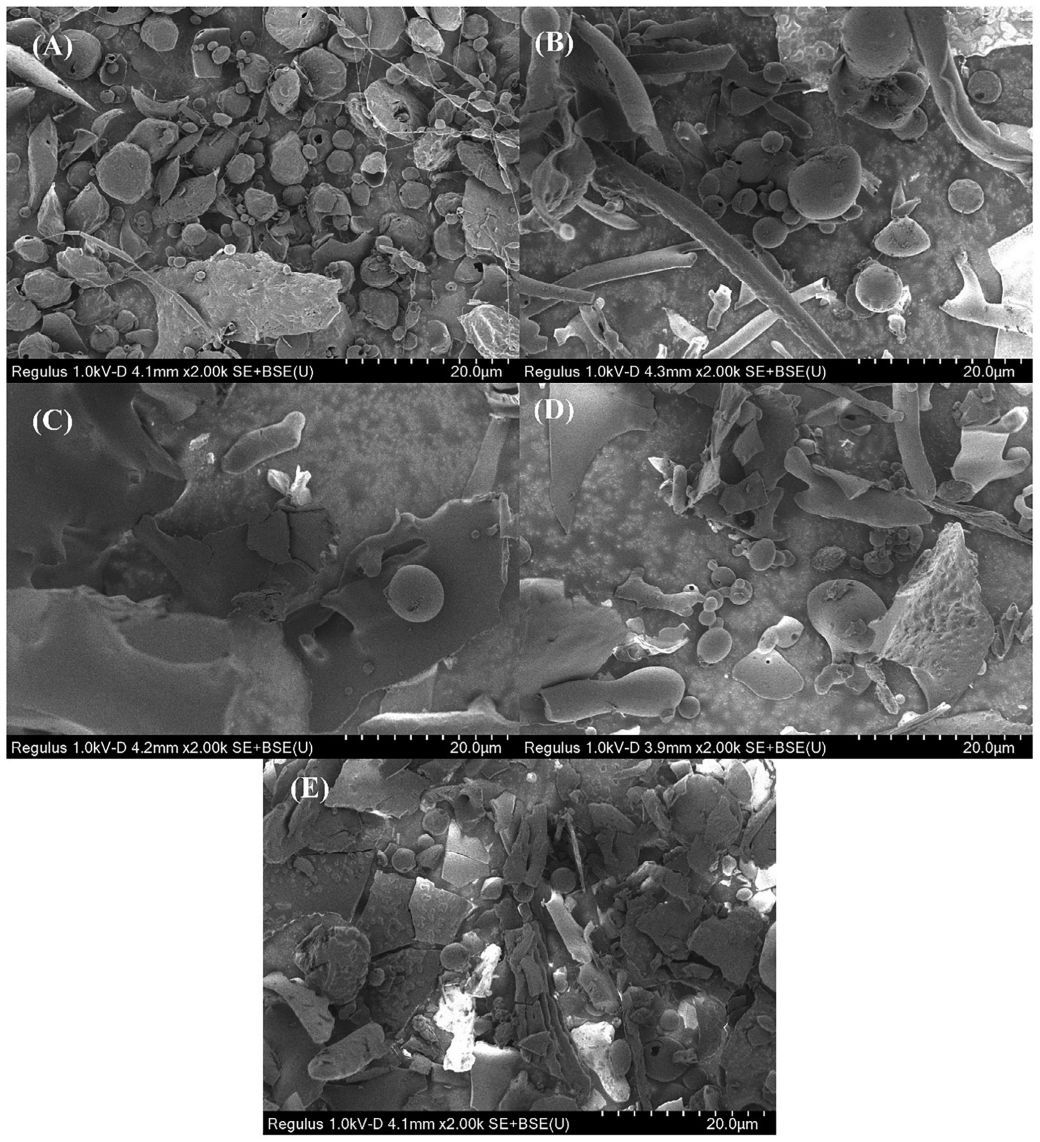
The SEM pictures of Bm‐CMP (A), Bm‐sCMP‐1 (B), Bm‐sCMP‐2 (C), Bm‐sCMP‐3 (D), and Bm‐sCMP‐4 (E). Bm‐CMP, *B. mori*‐hosted *C. militaris* polysaccharides; Bm‐sCMP‐1∼4, different sulfated Bm‐CMP derivatives.

### 2.4 Antibacterial Activity

To lay the foundation for the development of novel polysaccharide‐based formulations through structural modification, this study compared the antibacterial activities of native *B. mori*‐hosted *C. militaris* polysaccharides (Bm‐CMP) and their sulfated derivatives (Bm‐sCMP‐1∼4). Figure 6A,B depicted the ΔOD_600nm_ values of *Escherichia coli* (ATCC 25922) and *Bacillus subtilis* (ATCC 6633) treated with various samples. The results demonstrated a concentration‐dependent reduction in ΔOD_600nm_, indicating dose–responsive growth inhibition. Among the tested compounds, Bm‐sCMP‐2 exhibited the strongest antibacterial activity, with an MIC value of 2000 µg/mL against both bacterial strains. In contrast, Bm‐sCMP‐3 and Bm‐sCMP‐4 showed moderate activity, with MIC values of 4000 µg/mL for both strains, while Bm‐CMP and Bm‐sCMP‐2 displayed weak efficacy, with MIC values exceeding 8000 µg/mL. All these findings suggest that sulfation significantly enhanced the antibacterial activity of Bm‐CMP, and the effect was correlated with the DS. However, the relationship between the DS and antibacterial activity is not simply monotonic; excessive DS often leads to diminished efficacy, giving rise to a bell‐shaped activity profile [41]. This phenomenon was further explained by the Pearson correlation analysis. Furthermore, to gain deeper insights into the antibacterial mechanisms and delineate their antibacterial spectrum more comprehensively, future studies will expand on the current work by measuring inhibition zones, determining specific inhibition rates, and including a more diverse array of test bacterial species [42].

**FIGURE 6:**
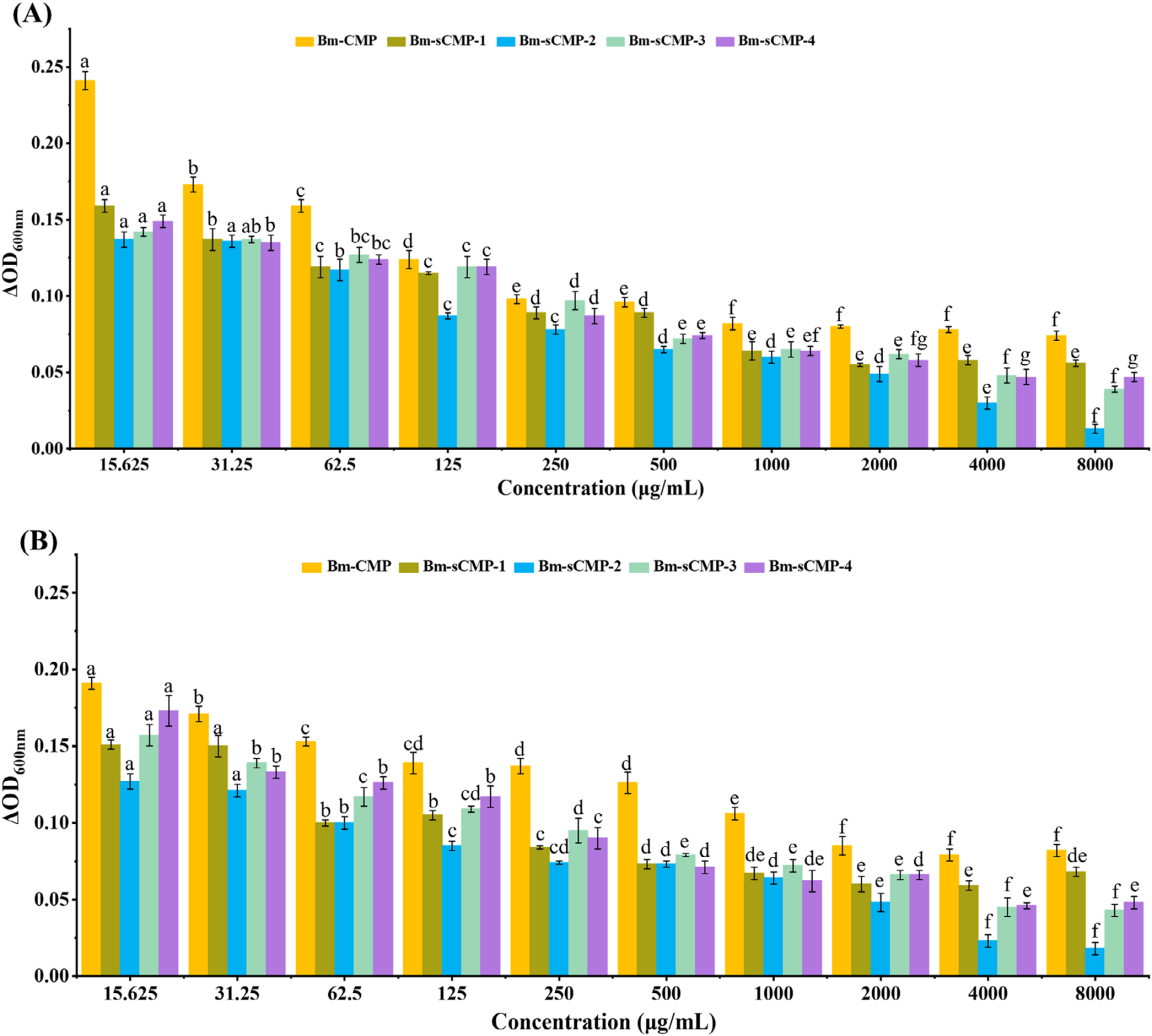
The antibacterial activity of Bm‐CMP and Bm‐sCMP‐1∼4 against *E. coli* (A) and *B. subtilis* (B), as well as the comparison of MIC at 2000 µg/mL. Different English letters indicate statistical differences (*p* < 0.05 or *p* < 0.01). Bm‐CMP, *B. mori*‐hosted *C. militaris* polysaccharides; Bm‐sCMP‐1∼4, different sulfated Bm‐CMP derivatives.

### 2.5 Correlation Analysis

Pearson correlation analysis was performed to preliminarily explore the structure–activity relationships (SAR) between the physicochemical properties and antibacterial efficacy of Bm‐CMP and its sulfated derivatives, with correlation coefficients spanning from −1 to 1 (Figure 7). The sulfate content exhibited a significant negative correlation with the MIC values against *E. coli* and *B. subtilis* (*p* < 0.05), preliminarily confirming that sulfation is the primary factor enhancing antibacterial efficacy. As shown in Table 1, all sulfated derivatives possessed significantly more negative zeta potentials than native Bm‐CMP, and this trend corresponded with the sulfate content. At the physical level, this modification imparts a strong anionic character to the polysaccharides, enabling efficient binding to positively charged bacterial membranes via electrostatic attraction, which serves as the driving force for membrane disruption, thereby enhancing the antibacterial activity of the polysaccharides [43, 44, 45]. Notably, the relationship between sulfate content and zeta potential was not strictly linear. The magnitude of the negative zeta potential (Table 1) plateaued as the sulfate content increased, indicating that excess sulfate groups are partly shielded by electrostatic repulsion and thus do not fully contribute to membrane binding [46]. This phenomenon further explains why Bm‐sCMP‐2 achieves the optimal balance between sulfate content and surface charge density, thereby exhibiting the superior antibacterial activity.

**FIGURE 7:**
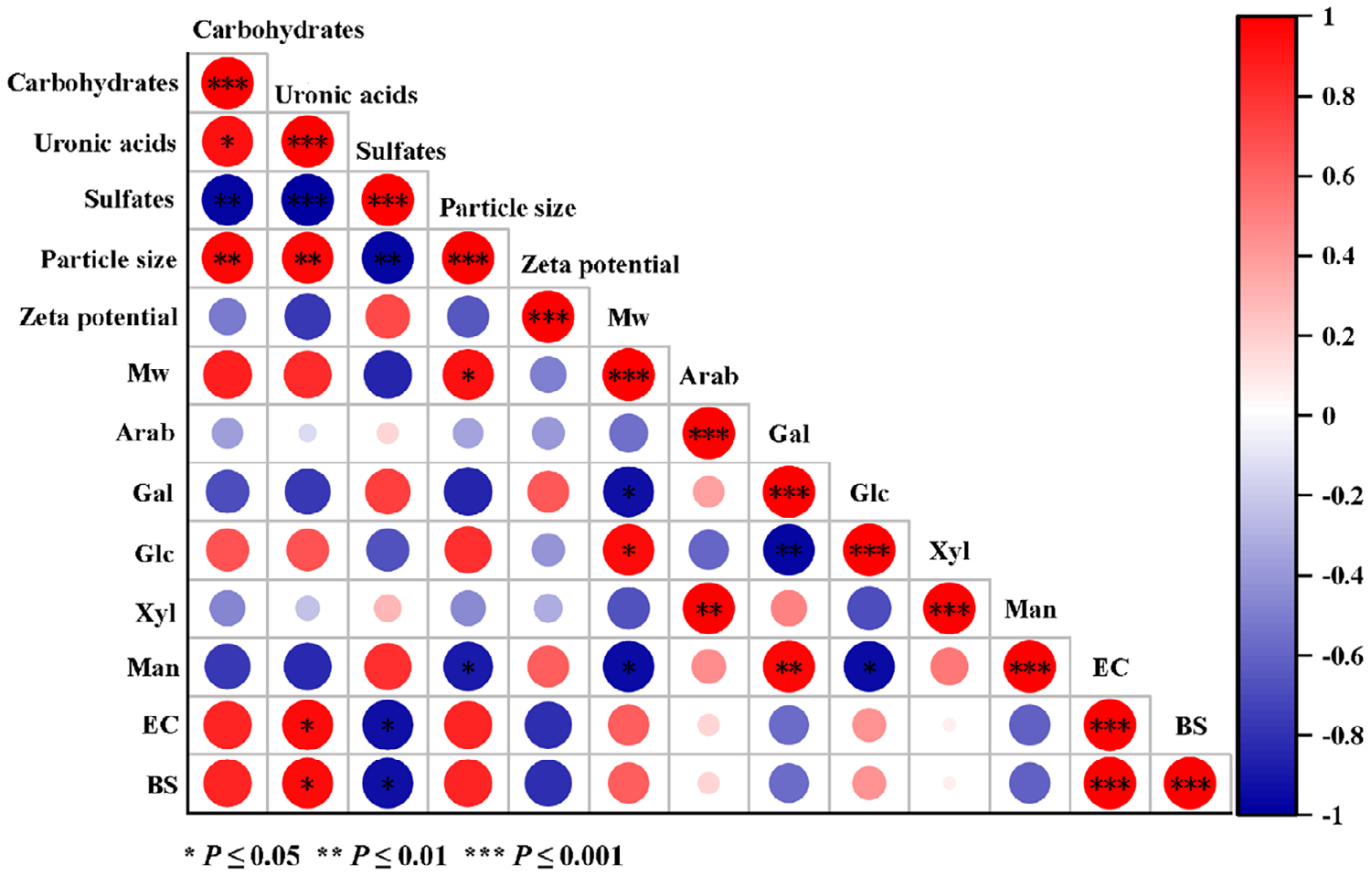
Pearson correlation analysis between physicochemical properties and antibacterial activity. Red symbols indicate positive correlations, whereas blue symbols denote negative correlations. Abbreviations: Mw, molecular weight; Arab, Arabinose; Gal, Galactose; Glc, Glucose; Xyl, Xylose; Man, Mannose; EC, *E. coli*; BS, *B. subtilis*.

In addition, uronic acid content showed a positive correlation with MIC (*p* < 0.05), which initially appears to contradict the conventional expectation that negatively charged carboxyl groups promote antibacterial activity [47, 48]. However, this phenomenon is primarily attributable to the fact that sulfation preferentially targets the sugar chain regions rich in uronic acids. This reaction substitutes their hydroxyl groups with sulfate groups, consequently reducing the relative uronic acid content [49]. Therefore, the positive correlation between uronic acid and MIC does not imply that uronic acids themselves inhibit bacterial growth. Instead, it merely reflects the structural alterations induced by sulfation. In this system, sulfate groups, rather than carboxyl groups, serve as the primary source of both negative surface charge and bioactivity. Nevertheless, when the DS becomes excessively high, essential hydroxyl groups are over‐substituted. This deprives the polysaccharide of the hydrogen bond donors and acceptors critical for target recognition, which may in turn diminish its bioactivity [50].

The Mw and particle size did not show significant correlations in this study, but this does not imply that Mw is irrelevant. Previous literatures have reported inconsistent findings regarding the relationship between Mw and antibacterial activity: some studies indicate that higher‐Mw polymers possess superior activity, whereas others suggest that lower‐Mw fractions are more potent due to better solubility and higher degrees of substitution [51, 52]. The lack of a significant correlation in our work likely indicates that, within the current series of sulfated derivatives, variations in sulfate content and zeta potential dominate the activity differences, while the contribution of Mw is relatively secondary. Furthermore, previous studies have demonstrated that polysaccharide nanosizing can significantly enhance antibacterial efficacy and confer long‐lasting activity. Smaller particle sizes provide a larger specific surface area, facilitating more thorough contact between the polysaccharide and bacterial membranes. Particle size may also affect the solubility and zeta potential of the polysaccharide in solution [53, 54, 55]. In our sulfated derivatives, particle size was negatively correlated with sulfate content (*p* < 0.05), suggesting that sulfation may induce molecular degradation and conformational changes. These structural alterations influence the aggregation state and effective particle size in aqueous media, thereby modulating the efficiency of bacteria‐polysaccharide interactions [40].

Monosaccharide composition is another structural parameter affecting bioactivity. Figure 7 shows that the correlations of Arab, Gal, Glc, Xyl, and Man with MIC were not statistically significant. However, previous studies have reported that sulfation may preferentially occur at specific hydroxyl positions (e.g., C‐6) on particular monosaccharide residues [56]. Differences in sulfation sites and degrees among different monosaccharide units could lead to distinct bioactivity [57]. The changes in monosaccharide relative proportions across derivatives (Table 1) reflect the site preference of sulfation, which may determine the efficiency of active site formation. For instance, the association between galactose content and activity might stem from its structural propensity for facile sulfation at specific sites [27, 58].

In summary, the antibacterial activity of Bm‐CMP sulfated derivatives is not governed by a single factor, but rather results from the synergistic interplay of multiple structural parameters. Among these, sulfate content plays a dominant role. It enhances electrostatic binding to bacterial membranes by increasing the surface negative charge. However, this effect operates within an optimal range, and Bm‐sCMP‐2 achieves this optimal balance. Excessive sulfation leads to increased steric hindrance, impaired chain conformation, and possibly polymer degradation, all of which attenuate activity. Meanwhile, Mw and particle size act as secondary factors, influencing the contact efficiency between the macromolecule and bacterial surfaces. Furthermore, monosaccharide composition suggests that the distribution pattern of sulfate groups along the sugar chain may be relevant to the formation of active sites. The Pearson correlation analyses statistically support these conclusions, providing a basis for future in‐depth studies on the SAR of such polysaccharides.

## 3 Conclusions

Four sulfated derivatives of polysaccharides from *B. mori‐*hosted *C. militaris* (Bm‐sCMP‐1∼4) with varying DS were successfully synthesized using Bm‐CMP as the precursor, aiming to investigate their physicochemical properties and antibacterial activities. The structures of Bm‐CMP and Bm‐sCMP‐1∼4 were characterized by various analytical methods, including general components, *ξ*‐potential, particle size, Mw, monosaccharide composition, FT‐IR, and SEM. The results preliminarily confirmed that sulfate groups were successfully introduced into Bm‐CMP. Additionally, sulfation was accompanied by partial molecular degradation and alterations in chain conformation, as evidenced by the reduction in Mw, changes in monosaccharide composition, and variations in particle size and surface morphology. Notably, sulfation significantly enhanced the antibacterial activity of Bm‐CMP in a DS‐dependent manner, with Bm‐sCMP‐2 demonstrating the strongest inhibitory effect. Pearson correlation analysis further revealed significant associations between key structural parameters and antibacterial efficacy, providing valuable references for preliminary SAR studies. Overall, these findings demonstrate that sulfation is a simple yet effective method for modulating the physicochemical properties and enhancing the bioactivity of natural polysaccharides. However, further research still needs to be conducted in the following aspects: (1) Broader clinical strains will be included to define the antimicrobial spectrum of Bm‐CMP and its sulfated derivatives, while mechanistic assays (membrane permeability, SEM, ROS, and leakage) will clarify how sulfation potentiates bactericidal activity. (2) More in‐depth separation procedures and structural characterization, such as NMR spectroscopy (^1^H and/or ^1^ ^3^C NMR), should be carried out to clarify its spatial conformation, deepening the understanding of the underlying SAR and exploring thoroughly the mechanism of antibacterial activity. (3) Future studies must also evaluate biocompatibility and toxicity by using in vitro skin cell models and in vivo animal models to ensure safety and therapeutic potential, thereby offering crucial insights into the development of topical formulations such as Bm‐CMP‐based sprays or hydrogels for dermatological applications.

## 4 Experimental Section

### 4.1 Materials and Reagents

The *B. mori*‐hosted *C. militaris* were procured from Suzhou Xinfudi Agricultural Products Market Development Co., Ltd. (Changshu, Jiangsu Province, China). All other experimental reagents and materials were of analytical grade and obtained from domestic suppliers.

### 4.2 Preparation of Bm‐CMP

The *B. mori‐*hosted *C. militaris* were dried at 75°C to constant weight, ground, and sieved through a 60‐mesh screen to obtain the powder, which was then defatted and reserved for subsequent experiments. After being weighed and soaked in water at a liquid‐to‐solid ratio of 1:30 (w/v) for 4 h, the processed samples were heated at 70°C for 3 h in a heating mantle. The extraction mixture was filtered after cooling, and then the filtrate was concentrated under reduced pressure to one‐fourth of its original volume. Three volumes of absolute ethanol were added, and the mixture was stored at 4°C for 24 h. The resulting precipitate was collected and lyophilized to obtain crude polysaccharides.

The yield of crude polysaccharides was calculated using Equation (1).

(1) $$Y\left(\%\right)=m/M\times 100\%,$$

where *m* represented the mass of the crude Bm‐CMP (mg), and *M* denoted the mass of the degreased powder obtained from *B. mori‐*hosted *C. militaris* (mg).

Deproteinization was carried out using a modified Sevag method. Briefly, the crude polysaccharide solution was sequentially treated three times with Sevag reagent (a mixture of chloroform and *n*‐butanol at a 4: 1, v/v ratio) [59]. After removing the residual Sevag reagent, the solution was dialyzed against distilled water for 72 h using a membrane with a Mw cut‐off (MWCO) of 3.5 kDa, with the external water replaced every 12 h. Finally, the retentate was collected and lyophilized to yield the deproteinized polysaccharides.

An appropriate amount of the deproteinized polysaccharide was dissolved in deionized water and applied onto a diethylaminoethyl (DEAE)‐cellulose‐52 column (2.6 × 60 cm) [60]. The column was eluted with 0.5 mol/L NaCl solution at a flow rate of 2 mL/min. The eluate was collected, concentrated under reduced pressure, and dialyzed against distilled water for 72 h (with water changes every 12 h) to remove salts. The dialyzed solution was then freeze‐dried to obtain the purified Bm‐CMP.

### 4.3 Sulfated Modification of Bm‐CMP

Sulfated Bm‐CMP derivatives were synthesized by a modified sulfation reaction. Briefly, Bm‐CMP (1.0 g) was dispersed in anhydrous *N*, *N*‐dimethylformamide (DMF), followed by the addition of sulfamic acid (SA, 2.0–5.0 g) and using urea as a catalyst. The mixture was first stirred at 60°C for 1 h to ensure homogeneity, and then heated at 80°C for 2 h. After cooling, the reactions were quenched with 10% NaOH solution, followed by ethanol precipitation at 4°C for 12 h [61]. The resulting precipitate was dialyzed against distilled water three times and then lyophilized to yield Bm‐sCMP‐1, Bm‐sCMP‐2, Bm‐sCMP‐3, and Bm‐sCMP‐4 (corresponding to Bm‐CMP: SA mass ratios of = 1:2, 1:3, 1:4, and 1:5, respectively). The sulfate content was quantified by the barium chloride–gelatin method [62] using a standard curve of *Y* = 0.586*X* − 0.0023, *R* ^2^ = 0.9988, and the DS was calculated according to Equation (2).

(2) $$\mathrm{DS}=\left(1.62\times S\%\right)/\left(32-1.02\times S\%\right),$$

where S% represents the percentage content of sulfates.

### 4.4 Characterization Methods

#### 4.4.1 Component Analysis

Except for sulfate and DS, the quantification of carbohydrates, proteins, and uronic acids in Bm‐CMP and Bm‐sCMP‐1 ∼ 4 was performed using three distinct colorimetric methods. For carbohydrate content, the phenol–sulfuric acid method [63] was employed with glucose as the standard, yielding the calibration curve: *Y* = 13.054*X* + 0.029 (*R* ^2^ = 0.9985).

Protein content was quantified by the Coomassie Brilliant Blue method [64] using bovine serum albumin (BSA) as the standard, giving the equation: *Y* = 5.9914*X* + 0.0221 (*R* ^2^ = 0.9953).

Uronic acid content was determined via the *m*‐hydroxybiphenyl method [65] with galacturonic acid as the reference, producing the calibration curve: *Y* = 5.4486*X* + 0.0189 (*R* ^2^ = 0.9962).

In all case, *Y* represented the absorbance and *X* denoted the concentration of the corresponding standard (mg/mL).

### 4.5 Particle Size and Zeta Potential

Solutions of Bm‐CMP and Bm‐sCMP‐1∼4 were prepared in deionized water at a concentration of 1.0 mg/mL. Prior to measurement, the solutions were filtered through a 0.45 µm membrane to remove dust and impurities. The particle size and zeta potential were then measured using a Zetasizer Nano ZS instrument (Malvern Instruments Ltd., Malvern, UK) equipped with dynamic light scattering (DLS) and electrophoretic light scattering (ELS) modules. All measurements were performed at 25°C.

### 4.6 Molecular Weight

The Mw distribution was characterized by high‐performance size‐exclusion chromatography (HPSEC) coupled with a refractive index detector (RID). The system comprised a Waters 1525 HPLC pump, a Waters 2414 RID detector, and the Empower 3 data processing workstation. Separation was performed on a Waters Ultrahydrogel Linear column (7.8 × 300 mm, 2 µm) maintained at 45°C. The mobile phase consisted of 0.1 M NaNO_3_, delivered at a flow rate of 0.9 mL/min. Prior to analysis, each polysaccharide sample was dissolved in ultrapure water at a concentration of 2.0 mg/mL and filtered through a 0.45 µm membrane. A third‐order calibration curve was established using a series of dextran standards with known Mws. The Mws of the polysaccharides were calculated based on the relationship between the retention time and the logarithm of the Mw [66].

### 4.7 Monosaccharide Composition

The monosaccharide composition was determined by ion chromatography following acid hydrolysis. Briefly, each sample (5.0 mg) was accurately weighed, mixed with 1 mL of 2 M trifluoroacetic acid (TFA) in a glass vial, and hydrolyzed at 121°C for 2 h. After cooling, the solution was diluted to 50 mL with ultrapure water, filtered through a 0.45 µm membrane filter, and analyzed immediately. Chromatographic separation was performed on a Dionex ICS‐5000^+^ system (Thermo Fisher Scientific, USA) equipped with a pulsed amperometric detector (PAD) and a CarboPac PA20 column (3 × 150 mm, 6.5 µm) maintained at 30°C. A gradient elution program was applied using eluent A (H_2_O), eluent B (250 mM NaOH), and eluent C (1 M sodium acetate) at a flow rate of 0.5 mL/min. The gradient profile was as follows: 0–21 min, 98% A and 2% B; 21.1–30 min, 93% A, 2% B, and 5% C; and 30.1–50 min, 20% A and 80% B. The injection volume was 20 µL [67].

### 4.8 Spectral Analysis

The UV spectra of each sample were recorded on a Persee T600 UV spectrophotometer (Puxi General Instrument Co., Ltd., Beijing, China) over the wavelength range of 200∼800 nm. All samples were prepared in deionized water at a concentration of 0.5 mg/mL.

For Fourier‐transform infrared (FT‐IR) spectroscopy, dried samples were mixed with KBr powder in an agate mortar and ground into a homogeneous fine powder. The mixture was pressed into a pellet using a hydraulic press, and FT‐IR spectra were recorded on an FTIR‐650 spectrometer (Gangdong Sci. & Tech. Co., Ltd., Tianjin, China) within a wavenumber range of 4000–400 cm^−1^ [68].

### 4.9 Morphological Analysis

Dried samples were mounted on silicon wafers and sputter‐coated with a thin layer of gold using an ion sputter coater (E‐1010, Hitachi). The surface morphology of Bm‐CMP and Bm‐sCMP‐1∼4 was examined by SEM (Regulus 8100, Hitachi High‐Tech Corporation, Tokyo, Japan) under vacuum conditions at an accelerating voltage of 1.0 kV and a magnification of 1.0k ×.

### 4.10 Antibacterial Activity

Bm‐CMP and Bm‐sCMP‐1∼4 were dissolved in sterile water to prepare stock solutions at a concentration of 16 mg/mL. In a sterile 96‐well plate, 100 µL of beef extract peptone medium (BPM) was mixed with 100 µL of the respective polysaccharide solution. Serial twofold dilutions were performed, resulting in final test concentrations of 8000, 4000, 2000, 1000, 500, 250, 125, 62.5, 31.25, and 15.625 µg/mL, with each concentration tested in triplicate. Subsequently, 100 µL of freshly prepared bacterial suspension in the logarithmic growth phase of *E. coli* (ATCC 25922, Gram‐negative) or *B. subtilis* (ATCC 6633, Gram‐positive) was added to the corresponding wells. The optical density at 600 nm (OD_600_) was immediately measured using a microplate reader to record the initial absorbance. The plate was then incubated at 37°C for 24 h, after which the OD_600_ was measured again. The change in absorbance (ΔOD_600_) was calculated by subtracting the initial value from the final reading [69]. The minimum inhibitory concentration (MIC) was defined as the lowest polysaccharide concentration at which ΔOD_600_ ≤ 0.05, indicating significant inhibition of bacterial growth.

### 4.11 Statistical Analysis

All measurements were carried out in triplicate, and the data were reported as mean ± standard deviation (SD). A one‐way analysis of variance (ANOVA) was performed using GraphPad Prism software (version 9.0) to assess statistical significance, with statistical significance defined as *p* < 0.05. Pearson's correlation analysis was performed using OriginPro 2025 to evaluate the relationships between variables.

## Author Contributions

**Jingchun Yang**: conceptualization, investigation, methodology, supervision and writing – original draft. **Jiani Cheng**: resources, investigation and writing – original draft. **Gaoyang Li**: resources, methodology and validation. **Wenpu Shi**: methodology and validation. **Yaqi Liu**: conceptualization and methodology. **Tingyu Luo**: funding acquisition. **Lixue Zheng**: writing – review, project administration and editing.

## Conflicts of Interest

The authors declare no conflicts of interest.

## Data Availability

The data supporting the findings of this study are available from the corresponding author upon reasonable request. These data are not publicly available due to privacy or ethical restrictions.
